# “Tianeptine abuse via novel, extended-release, star-shaped, drug delivery device”

**DOI:** 10.1016/j.toxrep.2023.07.006

**Published:** 2023-08-01

**Authors:** Henrik Galust, Justin A. Seltzer, Jeremy R. Hardin, Nathan A. Friedman, Alicia Minns

**Affiliations:** UCSD Fellowship in Medical Toxicology, Department of Emergency Medicine, UCSD Medical Center, 200W. Arbor Dr. #8676, San Diego, CA 92103, USA

**Keywords:** Tianeptine, Stablon, Coaxil, Tatinol, Delayed-release Capsule

## Abstract

We report a rare domestic case of exposure to tianeptine and use of a novel, extended-release, six-armed, star-shaped, drug delivery capsule. A 40-year-old male with a history of depression, anxiety, ethanol, opioid, cannabis, and tobacco use disorders presented to the emergency department (ED) from a substance abuse residential recovery treatment program after developing hypertension, tachycardia, and tremor for two day. He used an extended-release, six-armed, star-shaped, drug delivery device he purchased online, filling each arm with 15 mg of tianeptine (90 mg total). His intention was to mitigate the symptoms of kratom/opioid withdrawal through this extended-release method while simultaneously undergoing formal treatment for ethanol withdrawal. Tianeptine is an atypical tricyclic antidepressant that exerts complex mechanisms of action including serotonin (5-HT) neuromodulation as well as full μ-opioid and ∂-opioid receptor agonism. The capsule itself is made of caprolactone, which is a bioabsorbable material similar to absorbable sutures, initially developed as a long-term enteral antimalarial delivery method and is not FDA approved for human use. Over the course of the patients two day hospitalization course he developed symptoms consistent with uncomplicated ethanol withdrawal, which were treated with as-needed phenobarbital. No clinical manifestations of opioid or serotonin toxicity developed. Serial EKGs and telemetry monitoring remained unchanged. The patient was then medically cleared and discharged back to the residential recovery treatment program.

## Highlights

• Tianeptine is an atypical tricyclic antidepressant not approved in the US that exerts serotonin (5-HT) neuromodulation, μ-opioid, and ∂-opioid receptor agonism.
• Kratom has been used by patients as an alternative treatment for opioid use disorder.
• Delayed-release capsules made from biodegradable caprolactone may be purchased online and used by patients.

## Graphical Abstract

**Figure:**
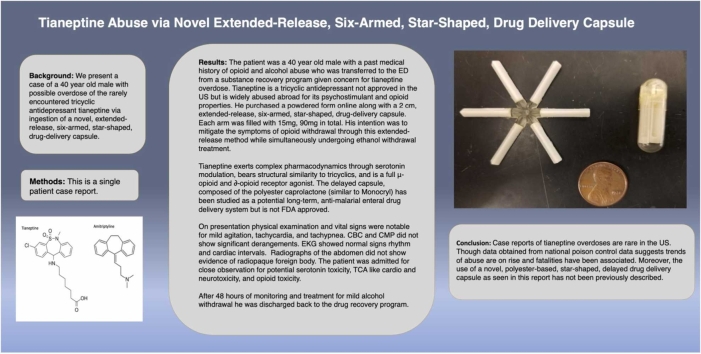

## 1 Introduction

Tianeptine, brand name Stablon, Coaxil, and Tatinol, is an antidepressant that is not approved by the United States Food and Drug Administration but is marketed widely across more than 60 countries [1]. Tianeptine is primarily prescribed to treat depression though it has been evaluated for several conditions such as bipolar disorder, dysthymia, and adjustment disorder [2], [3], [4]. Typical prescribed dosages range from 25 mg to 50 mg per day [4].

We report a rare domestic case of exposure to tianeptine and use of a novel, extended-release, six-armed, star-shaped, drug delivery capsule.

### 1.1 Case report

**Image 1:**
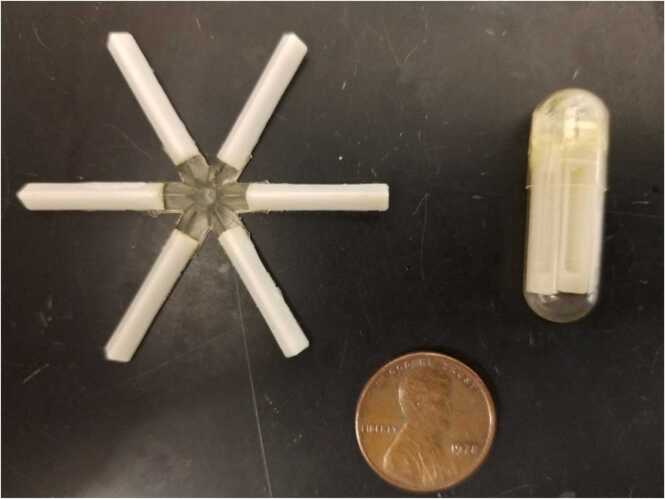
Star-shaped, polyester-based, delayed drug release capsule. Representative image courtesy of Robert Langer; Langer Labs MIT.

A 40-year-old male with a history of depression, anxiety, ethanol, opioid, cannabis, and tobacco use disorders presented to the emergency department (ED) from a substance abuse residential recovery treatment program after developing hypertension, tachycardia, and tremor two days after arrival there. The patient noted he had been using tianeptine the past four months in an effort to self-treat kratom abuse. At the time of presentation he had used an extended-release, six-armed, star-shaped, drug delivery device he purchased online (Image 1), filling each arm with 15 mg of tianeptine (90 mg total). His intention was to mitigate the symptoms of kratom/opioid withdrawal through this extended-release method while simultaneously undergoing formal treatment for ethanol withdrawal. He denied intentional self-harm or other co-ingestion. The last dose of the tianeptine containing drug delivery capsule was two days prior to presenting to the treatment facility, or approximately four days prior to ED presentation.

Initial vital signs were blood pressure 166/117 mmHg, heart rate 111 beats per minute, respiratory rate 18 breaths per minute, peripheral oxygen saturation 97%, and temperature 98.1°F. The patient was anxious appearing, notably with tachypnea and asterixis but had an otherwise unremarkable exam; no nystagmus, tongue fasciculations, hyperreflexia, clonus, diaphoresis, or mental status changes were noted. .

**Image 2:**
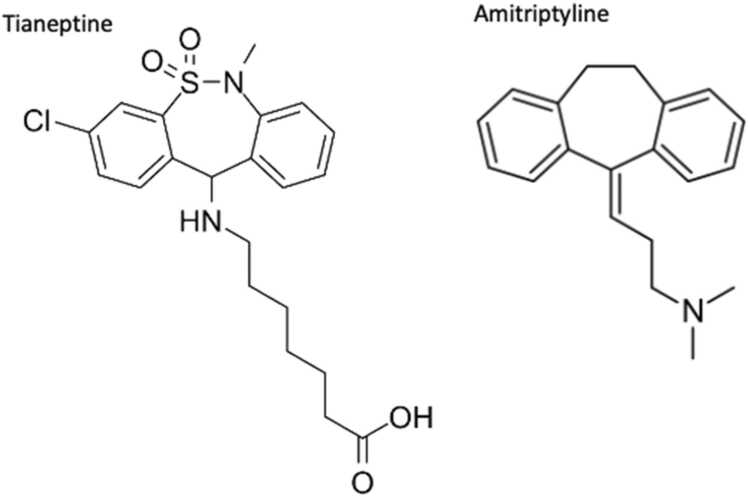
Structural similarity shared by tianeptine and tricyclic antidepressant amitriptyline.

Laboratory testing was significant for platelets of 128,000 cells/µL(150,00 – 450,000 cells/µL), AST 105 IU/L (5–35 IU/L), ALT 99 IU/L (5–35 IU/L). All other elements of the complete blood count and comprehensive metabolic panel were within normal limits. Serum ethanol concentration was undetectable. Urine drug screen was positive for barbiturates, benzodiazepines, and THC metabolites and negative for amphetamines, cocaine, methadone, opiates, and oxycodone. EKG showed normal sinus rhythm with normal cardiac intervals. Supine and upright radiographs of the abdomen did not show evidence of radiopaque foreign body.

Due to the structural similarity between tianeptine and tricyclic antidepressants, the toxicology service recommended close electrocardiographic and clinical monitoring for development of cardiotoxicity and manifestations of serotonin toxicity [5], [6], [7], [8]. As tianeptine is also known to have opioid effects, clinical monitoring for opioid toxicity was recommended as well [9], [10], [11], [12], [13], [14].

In regard to the delayed-release device, literature review of the capsule reported the device was composed of the biodegradable polyester polycaprolactone. It was designed to open within the stomach and abut the pylorus while slowly releasing medication for up to ten days [15]. As the patient had presented to the emergency department nearly 72 h post-ingestion, admission for continued observation for a period of at least 24 h was recommended due to his uncertain clinical trajectory.

During the patient’s subsequent two-day hospitalization, he developed symptoms consistent with uncomplicated ethanol withdrawal, which were treated with as-needed phenobarbital. No clinical manifestations of opioid or serotonin toxicity developed. Serial EKGs and telemetry monitoring remained unchanged. Serological studies were within normal studies. The patient was then medically cleared and discharged back to the residential recovery treatment program.

## 2 Discussion

The mechanism of action for tianeptine is thought to be multifactorial, though the primary therapeutic mechanism is thought to be due to serotonin (5-HT) neuromodulation, by decreasing 5-HT transmission through paradoxically increasing presynaptic reuptake of 5-HT [5], [6]. According to the manufacturer’s package insert tianeptine increases the rate of serotonin re-uptake by neuron in the cortex and hippocampus, increases spontaneous activity of pyramidal cells in the hippocampus, and accelerates neuronal recovery after functional inhibition [6]. Though tianeptine is structurally similar to tricyclic antidepressants (TCAs), reports of TCA-like toxicity are lacking. Volunteer studies and several case series of acute tianeptine ingestions have been published, none of which show TCA-like toxicity [16], [17], [18]. Additionally, tianeptine has full μ-opioid and ∂-opioid receptor agonist properties. Tianeptine’s opioid agonism has been implicated in potentiating dependency, abuse, and even death at supratherapeutic doses and several case reports have detailed recreational abuse [9], [10], [11], [12], [13], [14]. Our patient used tianeptine for its opioid agonist properties, specifically to “wean off of kratom.” Its abuse potential has led several states to make tianeptine a controlled substance, however as of this writing the United States Drug Enforcement Administration has not done so.

Regarding the oral capsule which the patient purchased online, few reports have been published regarding its in vivo effects. The capsule was initially developed by MIT researchers in 2016 with the intention of delivery of the antimalarial ivermectin via long-term enteral approach and has demonstrated effective delayed drug release up to 14 days in a swine model [15]. However, no human studies have been published and it is not approved for use in the United States. The capsule itself is made of caprolactone, which is a bioabsorbable material similar to absorbable sutures. Once ingested, the capsule deploys against the pylorus to allow for long-term delayed release until complete dissolution. Its shape allows for safe food passage [15]. To date, this innovative drug delivery method has not been approved by the FDA for human use. To our knowledge, our patient represents the first case report of drug abuse through via this novel delayed release device.

Our report is limited by the lack of confirmatory testing for both tianeptine and the delayed-release device. The patient's clinical presentation could have been entirely attributed to the effects of alcohol withdrawal. Nonetheless, this case represents a rare and noteworthy exposure.

## 3 Conclusion

Clinical experience with tianeptine in the United States is limited. Its online availability and diverse uses, including for its opioid effects, helps explain the rising rates of tianeptine abuse [19]. Use of the star-shaped delayed release capsule in this circumstance is, to our knowledge, novel. Further research regarding increased tianeptine abuse and utilization of this star-shaped capsule in this and other contexts is needed.

## Declaration of Competing Interest

The authors declare that they have no known competing financial interests or personal relationships that could have appeared to influence the work reported in this paper.

## Data Availability

No data was used for the research described in the article.
